# The immunomodulatory activity of lenvatinib prompts the survival of patients with advanced hepatocellular carcinoma

**DOI:** 10.1002/cam4.4312

**Published:** 2021-10-04

**Authors:** Jie Zhu, Peiqi Fang, Chong Wang, Meixiu Gu, Baishen Pan, Wei Guo, Xinrong Yang, Beili Wang

**Affiliations:** ^1^ Department of Laboratory Medicine, Zhongshan Hospital Fudan University Shanghai China; ^2^ Department of Laboratory Medicine, Xiamen Branch, Zhongshan Hospital Fudan University Xiamen China; ^3^ Department of Laboratory Medicine, Wusong Branch, Zhongshan Hospital Fudan University Shanghai China; ^4^ Department of Liver Surgery, Liver Cancer Institute, Zhong Hospital Fudan University Shanghai China

**Keywords:** CTL/Treg ratio, HCC, immunomodulatory activity, lenvatinib

## Abstract

**Background:**

Lenvatinib is a novel multiple receptor tyrosine kinase inhibitor used for hepatocellular carcinoma (HCC) treatment. Although its main function is to suppress VEGFR and FGFR pathway, its immunomodulatory activity in HCC is not elucidated. Thus, this study aimed to investigate the immunomodulatory capability of lenvatinib in HCC.

**Material and methods:**

Totally 47 patients with HCC were enrolled in this study, and the immune cells and serum cytokine profiles before initiation of treatment and after 1 and 3 months were measured. The immune checkpoint receptors on the immune cells were also evaluated. Kaplan–Meier survival estimate and log rank tests were used to assess the prognostic value.

**Result:**

The frequency of T helper (Th) cells and T regulatory (Treg) cells reduced after lenvatinib treatment, while cytotoxic T lymphocyte (CTL) cells increased significantly. The cytokine profiles showed IL‐2, IL‐5, IFN‐γ increased; other cytokines including IL‐6, IL‐10, TNF‐ α and TNF‐ β decreased with lenvatinib therapy. Furthermore, the PD‐1 and TIM‐3 expressed on CTL had greatly decreased; the expression of TIM‐3 and CTLA‐4 was reduced on Treg cells as well. Besides, the new index CTL/Treg ratio was created, and low ratio was associated with the unfavorable outcome of HCC patients.

**Conclusion:**

Our results confirmed that lenvatinib is capable of improving patients’ immune status, saving the effector cells from exhaustion status and inhibiting the number and function of immunosuppressive cells. The novel index CTL/Treg ratio qualifies as a predictor for the outcome of patients with lenvatinib therapy.

## Graphical abstract

The immunomodulatory activity of lenvatinib saves the effector cells from exhaustion status and inhibiting the number and function of immunosuppressive cells.

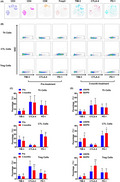

## 1 INTRODUCTION

Hepatocellular carcinoma (HCC) is one of the most common malignant tumors globally and remains as the fourth leading cause of cancer‐related death during the recent decade.^1^ The most effective and preferred curative treatment is surgical removal of tumor.^2^, ^3^ However, due to the insidious progression of that fatal disease, most patients were already in advanced stage at the time of diagnosis, missing the chance to receive curative resection.^4^, ^5^ Fortunately, benefited from the development of genetics and genomic technology, the landscape of molecular alternation of HCC was initially established, among which some critical driver mutations could lead to druggable targets.^6^ Lenvatinib is a such kind of molecule inhibitor of multiple receptor tyrosine kinases, which approved for the emerging first‐line therapy of advanced stage HCC in China.^7^, ^8^ In addition, it showed noninferiority in time to progression and overall survival (OS) compared with sorafenib in clinical trial.^9^ As reported, lenvatinib extends its antitumor and antiangiogenic activities through suppressing VEGF‐VEGFR and FGF‐FGFR system, which were considered as the most important factors in tumorigenesis and disease progression.^10^ On the other hand, immune modulatory effects also played a vital role in tumor development. Several research had already showed that when combined with immune‐checkpoint inhibitors, lenvatinib could greatly enhance its antitumor effect^11^, ^12^; other reports indicated lenvatinib itself has ability to alleviate the immune suppression^13^ and further affects the immune cells^14^ so as to improve the prognosis of patients with tumor burden. However, there is no relevant research explains how lenvatinib regulates the immune status of HCC patients.

So, in this study, our team conducted an retrospective research to evaluate the change of immune cells and relevant cytokines in patients with HCC after they received lenvatinib therapy.

## 2 MATERIAL AND METHODS

### 2.1 Enrollment and specimen collection

A total of 47 patients with unresectable HCC were enrolled in Zhongshan Hospital, Fudan University, from July 2020 to October 2020. The inclusion criteria were as follows: Patients were diagnosed with HCC according to the Guidelines for the Diagnosis and Treatment of Hepatocellular Carcinoma (2019 Edition)^15^ and received only lenvatinib treatment (8 mg/day for weight <60 kg; 12 mg/day for weight ≥60 kg). If no dose‐limiting toxicity (DLT) occurred in the first phase, the second phase would be initiated using the recommended dose from the DLT phase, and regimens may be changed and even discontinued with the occurrence of unacceptable or serious Adverse events. The exclusion criteria were as follows: (1) patients with history of other tumors; (2) patients had received treatments before admission; and (3) patients had 50% or higher occupation of liver; (4) patients with major diseases of other system, such as severe cardiovascular, lung diseases and neurological diseases, or mental disease. This research had been approved by the research ethics committee of Zhongshan Hospital, Fudan University. The written informed consent was obtained from every patient in this study. The detailed clinical characteristics are shown in Table 1.

**TABLE 1:** The clinicopathologic characteristics of patients with hepatocellular carcinoma enrolled in the study

Clinical characteristic	Number of patients
Age	
≤50	8
>50	39
Sex	
Male	41
Female	6
AFP	
≤400 ng/ml	10
>400 ng/ml	37
ALT	
≤75 U/L	18
>75 U/L	29
HbsAg	
Negative	6
Positive	41
ECOG	
0	11
1	36
Portal vessel invasion	
No	28
Yes	19
Child–Pugh score	
A	14
B	33
BCLC stage	
B	15
C	32

Abbreviations: AFP, alpha fetoprotein; ALT, alanine aminotransferase.

Two millilitre of blood anticoagulated with EDTA‐k_2_ and 2 ml of serum were collected from each patient with HCC before initiation of the treatment and after 1 and 3 months of the treatment with lenvatinib.

### 2.2 The follow‐up of patients with hepatocellular carcinoma

The observation period began from the start of therapy with lenvatinib to patients’ death or the last visit. Every patient was monitored by serum testing including alpha fetoprotein (AFP), alanine aminotransferase (ALT), aspartate aminotransferase, etc. and received imaging test including CT or MRI scans 4–6 weeks after the initiation of therapy and thereafter every 2–3 months. The patients were further divided into four groups, including complete response (CR) group, partial response (PR) group, stable disease (SD) group, and progressive disease (PD) group based on the prognosis according to the Response Evaluation Criteria in Solid Tumors (RECIST)1.1,^16^ and the RECIST result was the smallest sum, longest diameter recorded since the start of the treatment or the appearance of one or more new lesions. CR was defined as the disappearance of intratumoral arterial enhancement in any target lesions and no new lesion appearance; PR was defined as a ≥30% reduction in the sum of the diameters of target lesions; PD was defined as a ≥20% increase in the sum of the diameters of target lesions or appearance of new lesions; and SD was defined as any other case that did not qualify for either PR or PD. Progression‐free survival (PFS) was defined as the interval between the initiation of therapy and disease progression. OS was defined as the interval between the initiation of therapy and death. The treatment response rate for patients with HCC was shown in Table S1.

### 2.3 Flow cytometry

Peripheral blood mononuclear cells were isolated through Ficoll (GE Healthcare Bio‐sciences AB, Sweden) from whole blood of patients with HCC and resuspended in 0.5 ml of PBS. Then the immunofluorescent antibodies were deployed to further distinguish the different kinds of immune cells in peripheral blood. The immunofluorescent antibodies used in the study were shown as followed: CD3‐FITC, CD4‐APC, CD25‐PE‐Cy7, PD‐1‐BV421, Foxp3‐V450, CTLA‐4(CD152)‐PE, TIM‐3‐PerCP‐Cy5.5, CD8‐ APC‐Cy7, CD56‐PE, and CD19‐ PerCP‐Cy5.5 (BD Bioscience). The related isotype antibodies were used as negative control. The result was analyzed with Flowjo 10.6.2 (BD Bioscience).

### 2.4 Cytokines profiles

The cytokines including IL‐1β, IL‐2, IL‐4, IL‐5, IL‐6, IL‐8, IL‐10, IL‐12p, IL‐17a, IL‐17f, IL‐22, TNF‐ α, TNF‐β, and IFN‐γ were measured using CBA cytokine kit (QuantoBio). The measurement was followed by the manufacturer's protocol.

### 2.5 Statistical analysis

Statistical analyses were performed using SPSS 19.0 software (IBM). Experimental values were expressed in the form of mean ± SD. The difference between the groups was compared using Student *t*‐test, chi‐square test, or Fisher test, as appropriate. Kaplan–Meier survival curves were performed defining the survival rates, log‐rank test was used to access the different between the groups. *p* value <0.05 was considered as statistically significant.

## 3 RESULT

The change of immune cells in peripheral blood of patients with HCC after receiving lenvatinib treatment.

**FIGURE 1:**
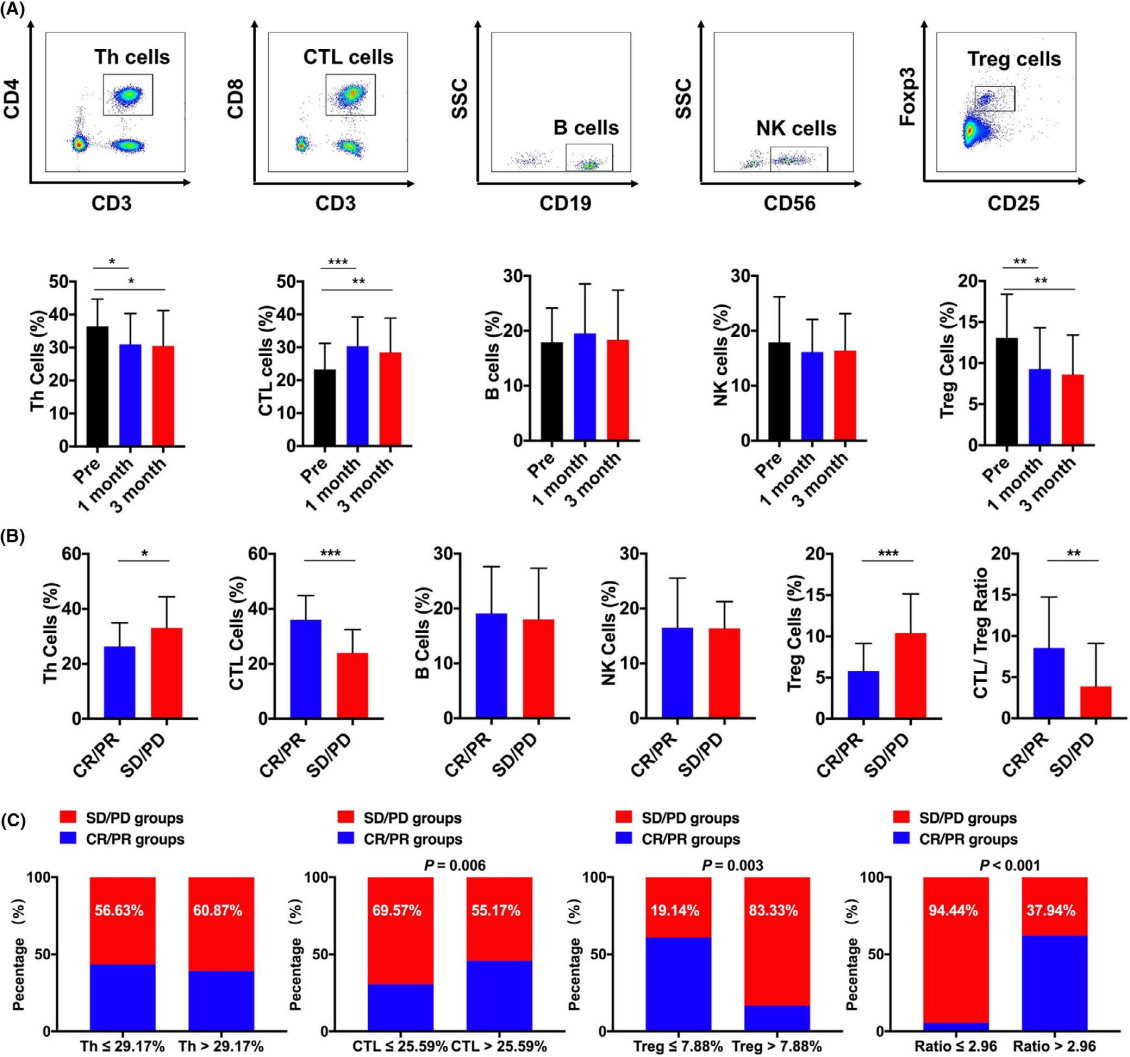
The change of immune cells from patients with HCC after lenvatinib therapy. (A) Frequency of CD4^+^ Th cells, CD8^+^CTL cells, Treg cells, B cells, and NK cells pretreatment and after 1 and 3 months. (B) The frequency of Th cells, CTL cells, B cells, NK cells, and CTL/Treg ratio in CR/ PR group and SD/ PD group. (C) The percentage of poor outcome in Th group, CTL group, Treg group, and CTL/Treg ratio group. * *p* < 0.05; ** *p* < 0.01; *** *p *< 0.001

The level of various kinds of circulating immune cells in patients with HCC was measured and evaluated by flow cytometry prior to lenvatinib therapy as well as 1 month (approximately 29–32 days) and 3 months (approximately 88–93 days) after the treatment. The result showed that the T helper cells (CD4^+^ Th cells) had slightly decreased with the process of lenvatinib treatment from 36.38 ± 8.36% to 31.02 ± 9.34% (1 month) (*p *= 0.015) and 30.46 ± 10.79% (3 months) (*p *= 0.031), whereas cytotoxic T lymphocyte cells (CD8^+^ CTL cells) had dramatically increased from 23.31 ± 7.92% to 30.36 ± 8.83% (1 month) (*p *= 0.001) and 29.28 ± 10.34% (3 months) (*p *= 0.003). Statistically significant reduction was also observed in the frequency of T regular (Treg) cells after 1 month of lenvatinib treatment (9.48 ± 5.31%) (*p *= 0.004) compared with its baseline values (13.42 ± 5.94%), and it kept falling during 3 months (8.94 ± 5.11%) (*p *= 0.003). However, no obvious change of B cells or natural killer (NK) cells was observed during the experiments (Figure 1A). Next, we further evaluate the frequency of these three types immune cells in patients with HCC with different clinical response, and the result indicated that after 3‐month treatment, the frequency of CTL cells was significantly elevated in CR and PR groups (35.95 ± 8.92%) (totally 18 patients) compared with those in SD and PD groups (23.93 ± 8.51%) (totally 29 patients) (*p *< 0.001). On the contrary, the frequency of Th (CR/PR:26.37 ± 8.52%; SD/PD:30.00 ± 11.38%; *p *= 0.039) and Treg cells (CR/PR:5.79 ± 3.33%; SD/PD:10.39 ± 4.74%; *p *< 0.001) was decreased in those patients with better prognosis (Figure 1B). Interestingly, when we combined CTL and Treg cells to form a new index (the ratio of CTL/Treg) based on the third‐month result, patients in CR and PR groups had much higher CTL/Treg ratio value (*p *= 0.008). We then stratified patients with HCC as CTL high and CTL low, Th high and Th low, Treg high and Treg low, and CTL/Treg ratio high and CTL/Treg ratio low based on the median of these factors. The result showed patients with high CTL/Treg ratio value (*p *< 0.001), high CTL (*p *= 0.006), and low Treg (*p *= 0.003) achieved better outcome, and the CTL/Treg ratio had the best efficacy, suggesting the new index had great ability in discriminating the different outcome of patients with HCC with lenvatinib treatment (Figure 1C).

### 3.1 Association of CTL/Treg ratio with clinicopathologic parameters and its clinical significance in patients with hepatocellular carcinoma

Next, we tried to seek the association between CTL/Treg ratio and clinicopathologic parameters in patients with HCC. The results showed that CTL/Treg ratio was related to AFP (*p *= 0.012), indicating patients with lower CTL/Treg ratio tended to have higher AFP. Meanwhile, patients in BCLC‐0‐A had little bit higher CTL/Treg ratio value than those in BCLC‐B‐D stage (*p *= 0.060), although it had not yet reached a statistical significance. Other clinical characteristics, including age, sex, ALT, HbsAg, portal vessel invasion, and Child–Pugh score had no significant difference between the high and low CTL/Treg ratio groups (Table 2).

**TABLE 2:** Correlation between CTL/Treg ratio and clinicopathologic characteristics

Clinical characteristic	Number of patients	CTL/Treg ratio ≤2.96	CTL/Treg ratio >2.96	*p*
Age				
≤50	8	5	3	0.461
>50	39	18	21	
Sex				
Male	41	21	20	0.666
Female	6	2	4	
AFP				
≤400 ng/ml	10	1	9	0.012
>400 ng/ml	37	21	16	
ALT				
≤75 U/L	18	8	10	0.763
>75 U/L	29	13	14	
HbsAg				
Negative	6	1	5	0.188
Positive	41	22	19	
Portal vessel invasion				
No	28	15	13	0.556
Yes	19	8	11	
Child–Pugh score				
A	14	5	9	0.341
B	33	18	`15	
BCLC stage				
0‐A	15	4	11	0.060
B‐D	32	19	13	

Abbreviations: AFP, alpha fetoprotein; ALT, alanine aminotransferase.

### 3.2 The prognostic value of immune cells and new index in patients with hepatocellular carcinoma treated with lenvatinib

**FIGURE 2:**
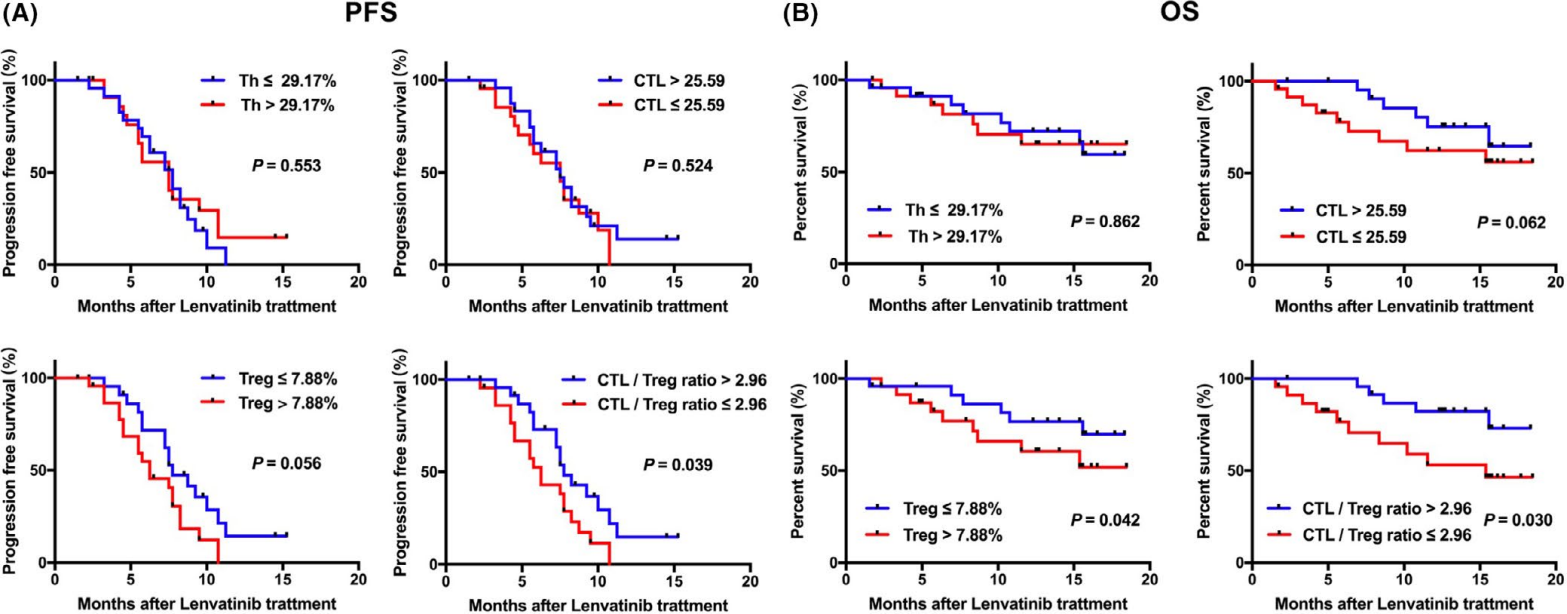
Kaplan–Meier survival analysis of patients with high‐ and low‐lymphocyte cells and the novel index in the two cohorts

Because we have found that CTL, Th, Treg cells, and CTL/Treg ratio had great difference in CR/PR and SD/PD groups, the prognostic value of these immune cells and the novel index were further exploded using Kaplan–Meier survival curve. The log‐rank result indicated high CTL/Treg ratio was associated with longer PFS (*p *= 0.039) and OS (*p *= 0.030) of patients with HCC undergoing lenvatinib treatment. Although low Treg correlated with longer PFS (*p *= 0.056) and OS (*p *= 0.030). In addition, high CTL cells had slight connection with better OS (*p *= 0.062), but it showed no significant prognostic value in PFS (*p *= 0.524), whereas Th cells had no prognostic significance for both PFS (*p *= 0.553) and OS (*p *= 0.862) (Figure 2A,B).

### 3.3 Serum cytokine profiles

**FIGURE 3:**
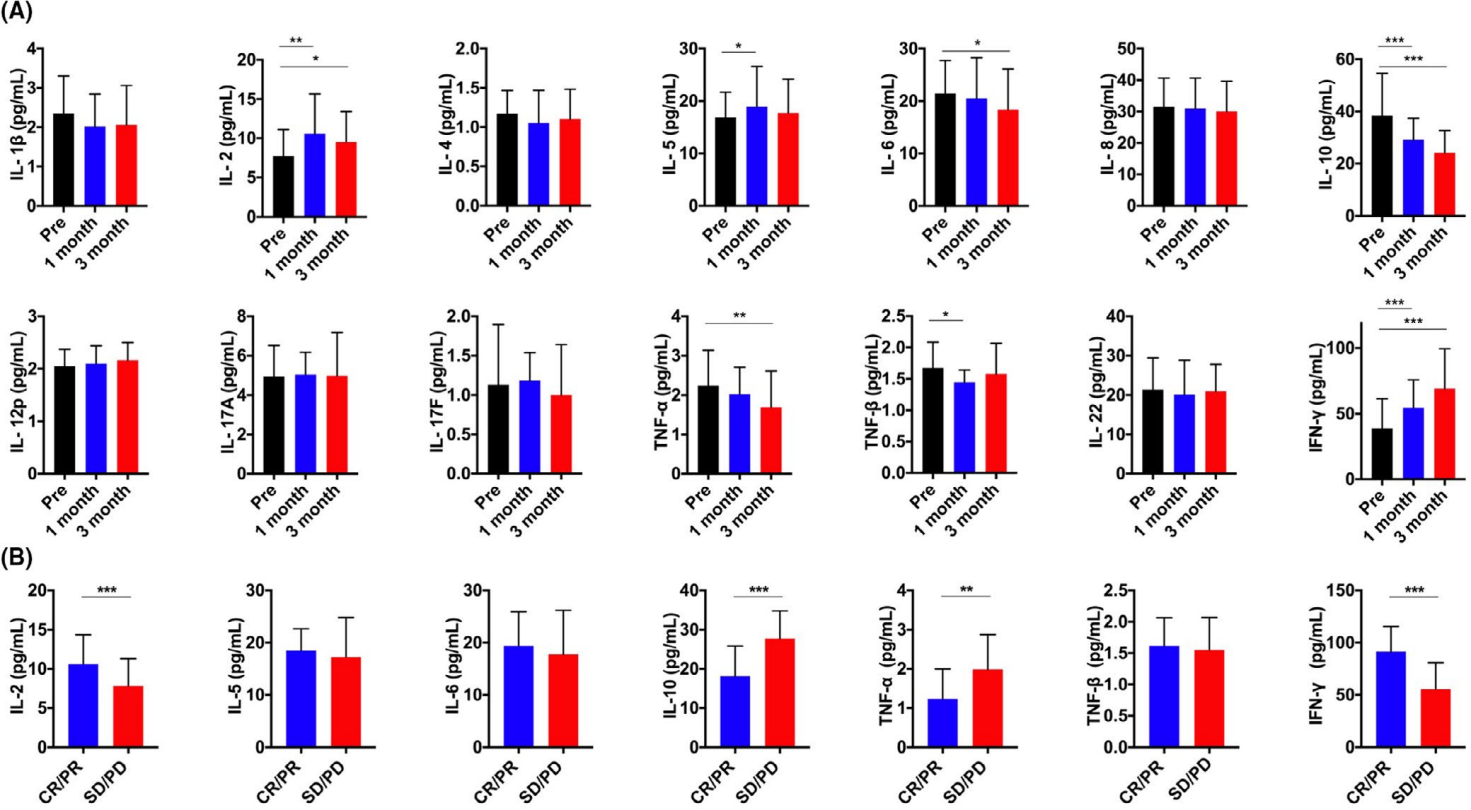
The change of 14 cytokines in the serum from patients with HCC after lenvatinib therapy. (A) The serum level of 14 cytokines pretreatment and after 1 and 3 months. (B) The serum level of 14 cytokines in CR/ PR group and SD/ PD group. * *p* < 0.05; ** *p* < 0.01; *** *p *< 0.001

We next evaluated the expression levels of relevant cytokines of these 47 patients with HCC before and after the treatment with lenvatinib during 3 months. The result showed that IL‐2 (1 month: *p *= 0.008; 3 months: *p *= 0.014), IL‐5 (1 month: *p *= 0.042), IFN‐γ (1 month: *p *= 0.001; 3 months: *p *< 0.001) increased, whereas several cytokines including IL‐6 (3 months: *p *= 0.038), IL‐10 (1 month: *p *= 0.001; 3 months: *p *< 0.001), TNF‐ α (3 months: *p *= 0.004), and TNF‐ β (1 month: *p *= 0.013) decreased, the remaining cytokines had no significant change (Figure 3A). We then further evaluated the expression level of those cytokine of statistically significance in patients with HCC with different clinical response. IL‐10 (*p* < 0.001) and TNF‐ α (*p *= 0.004) were significantly evaluated in SD/PD groups. Besides, IL‐2 (*p *= 0.001) and IFN‐γ (*p *= 0.001) were decreased in SD/PD groups compared with CR/PR groups. However, IL‐5, IL‐6, and TNF‐ β showed no difference between CR/PR groups and SD/PD groups (Figure 3B).

### 3.4 The expression level of immune checkpoint receptors on T cells

**FIGURE 4:**
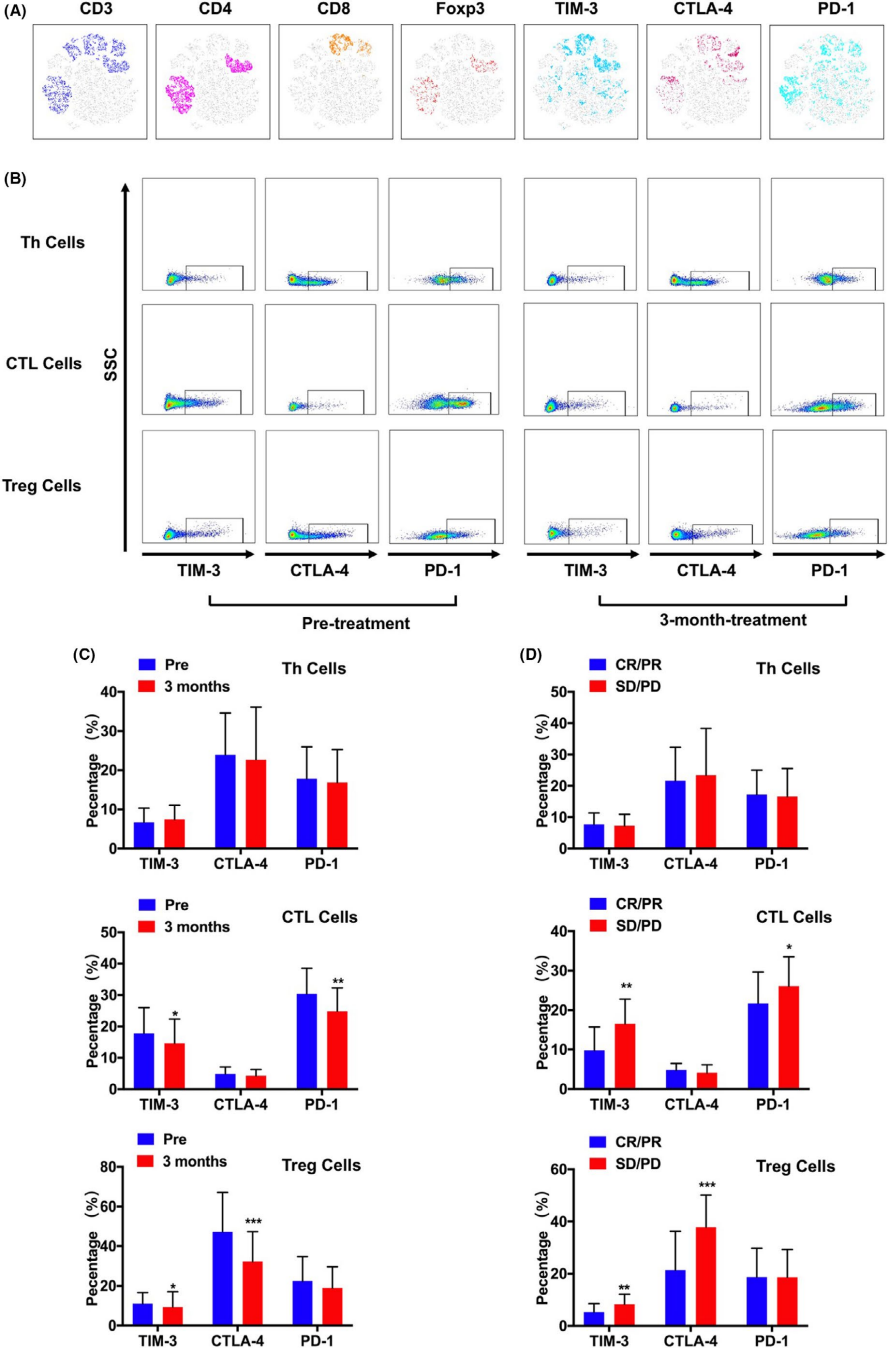
The expression of immune checkpoint receptors from immune cells of patients with HCC were measured by flow cytometry followed by viSNE analysis. (A) The expression of CD3, CD4, CD8, TIM‐3, CTLA‐4, and PD‐1 was indicated by the different color. (B) Representative flow cytometry result showing the expression level of TIM‐3, CTLA‐4, and PD‐1 on Th cells, CTLA cells and Tregs. (C) The change of immune checkpoint receptors from immune cells pretreatment and after 3 months. (D) The expression level of immune checkpoint receptors on Th cells, CTLA cells, and Tregs in CR/ PR group and SD/ PD group. * *p* < 0.05; ** *p* < 0.01; *** *p *< 0.001

viSNE based on flow cytometry was used to further analyzed the phenotypic characteristics of immune checkpoint receptor including programmed death 1 (PD‐1), cytotoxic T‐lymphocyte‐associated protein 4 (CTLA‐4), and mucin domain‐containing protein 3 (TIM‐3) on T cells. As shown in Figure 4A, PD‐1 was expressed most on CTL and Th cells, CTLA‐4 was expressed on Treg and Th cells, and TIM‐3 was expressed on CTL cells. After lenvatinib treatment, the PD‐1 (*p *= 0.008) and TIM‐3 (*p *= 0.015) expressed on CTL had significantly decreased. The reduction of TIM‐3 (*p *= 0.013) and CTLA‐4 (*p *= 0.001) was also observed on Treg cells. However, there was no great change of CTLA‐4 on Th cells (Figure 4B,C). Next, we further analyzed the expression of these immune checkpoint receptors in patients with HCC with different clinical response. Patients in CR/PR groups had lower expression of TIM‐3 (*p *= 0.007) and PD‐1 (*p *= 0.022) on CTL cells, additionally, the expression of CTLA‐4 (*p *< 0.001) and TIM‐3 (*p *= 0.006) on Treg in CR/PR groups was much lower compared with DS/PD groups. (Figure 4D).

## 4 DISCUSSION

HCC is one of the most common malignant tumors in the world, and its incidence and mortality rate are increasing annually, bringing heavy burden and waste to social medical resources.^17^ For the prognosis of patients with HCC, early diagnosis and treatment are always the major issue. Currently, liver resection, tumor ablation, and liver transplantation are commonly used treatments. In addition, with developing advancements including living donor transplantation and laparoscopic liver resection, new therapies provide more options for different patients and influence their long‐term survival.^18^, ^19^ In the past decade, systematic treatment attracted a great attention. Sorafenib was considered as the most important and effective first‐line medicine in treating advanced and unresectable HCC. However, the choice of first‐line treatment still remained limited because few agents showed non‐inferiority or superiority to sorafenib.^20^, ^21^

Lenvatinib is a selective, oral, small‐molecule inhibitor of multiple‐receptor tyrosine kinases. With different dosages, it can exert different inhibitory effects on a variety of pathways related to pro‐angiogenesis and tumorigenesis,^22^, ^23^, ^24^ among which VEGFR and FGFR are the main targets, which are overexpressed in tumor and have great connection with proliferation of cancer cells and angiogenesis. Several clinical trials demonstrated that lenvatinib could significantly prolong the median OS of patients with HCC, and it even showed better clinical efficacy than sorafenib.^8^, ^25^, ^26^ Moreover, as reported, when combined with immune checkpoint therapy, the prognosis of patients with HCC had been greatly improved.^11^, ^12^, ^27^ In addition, pervious research demonstrated that lenvatinib monotherapy could increase tumor‐infiltrating macrophages and CD8^+^ T cells through mice model in anaplastic thyroid cancer.^28^ However, whether lenvatinib could regulate or affect the immune status of patients with HCC had not been fully clarified so far. In this study, we first found that after lenvatinib therapy, the frequency of CD8^+^ T cytotoxic cells in peripheral blood of patients with HCC had continuously and significantly increased; meanwhile, those patients with higher CTL cells appeared to achieve better clinical response and OS. Pervious study showed that the immune escape of tumor cells is one of major mechanisms of tumor progression and metastasis, resulting from the exhaustion of CTL cells, which predominantly exert antitumor efficacy.^29^ A great number of studies also gave strong support that CTL cells, especially tumor‐infiltrating CTL cells, which have been associated with better prognosis and lower recurrence rates after proper treatment.^30^, ^31^, ^32^, ^33^ In detail, CTL cells have the capability to recognize respective tumor antigens in autologous HCC tissue, killing the target cells through cytotoxic effect.^34^ Recently, Kimura et al. had reported that lenvatinib showed more effective antitumor activity than sorafenib in immune‐competent mice and weakened by CTL cell depletion,^35^ suggesting lenvatinib's antitumor effect might be related to the number and viability of CTL cells, which interestingly was consistent with ours. Besides, we further explored the immune checkpoint receptors on CTL cells. As showed in Figure 4, PD‐1 and TIM‐3 was significantly upregulated on CTL cells before the initiation of treatment. Previous reports demonstrated that high expression level of PD‐1 on T cell was considered as a major marker of T cell progressive dysfunction.^36^ In addition, high level of TIM‐3 was confirmed as a negative regulator of T cells function.^37^ Apparently, CTL cells were in the status of exhaustion with high expression of TIM‐3 and PD‐1.^38^, ^39^ Sawada et al. thought that PD‐1^+^ Tim3^+^ CTL cells were a kind of immune cells that lost cytotoxic activity in cancer and associated with poor outcome.^40^ In our study, we found after therapy, the expression of PD‐1 and TIM‐3 on CTL cells had dramatically reduced. Moreover, those patients who had complete and partial response had lower PD‐1 and TIM‐3 expression, suggesting the CTL cells might recover from exhaustion status and form a new immune balance with lenvatinib treatment processing. Coincidently, the cytokine profiles were in favor of our hypothesis, after 3‐month treatment, IL‐2 and IFN‐γ were elevated continuously. IL‐2 is mainly secreted by CD4^+^ cells^41^ and considered as one of the strongest stimuli causing the proliferation and differentiation of naive CD8^+^ cells and formation of functional memory cells as well.^42^ In patients with HCC, high level of IL‐2 further activated CD8^+^ cells, promoting them to secrete IFN‐γ to enhance the efficiency of killing cancer cells. This phenomenon was consistent with what we had observed in the study.

On the contrary, CD4^+^ T helper cells had slightly decreased, whereas the frequency of Treg in peripheral blood showed great difference before and after medication. Multiple research had already confirmed that Treg is one of the most vital immunosuppressive cells in the progression of tumor, standing out for its ability to inhibit various kinds of immune cells through contact‐dependent interaction and cytokines it secrets including TGF‐ β, IL‐10, and IL‐35.^43^, ^44^, ^45^, ^46^ In the study, we stratified the patients with HCC into two groups based on the median of Treg frequency, the result showed those patients with lower Treg tended to achieve better outcome and longer OS. To our surprise, two patients’ Treg dropped significantly from 41% to 2% and 38% to 1.8% during the lenvatinib therapy, suggesting lenvatinib itself might bring some influence on Treg generation. Moreover, the immune checkpoint receptors on Treg were measured as well. We had noticed that after treatment the expression level of CTLA‐4 and TIM‐3 all decreased greatly. As reported, CTLA‐4 is a key molecule expressed on the surface of Treg, whose main function is to downregulate the costimulatory molecules CD80/86 expression in antigen‐presenting cells, thereby inhibiting the activation of effector T cells.^47^ Additionally, several studies demonstrated that TIM‐3 was identified as a novel marker of Treg cells that were highly effective at suppressing T cell proliferation despite of high expression of PD‐1.^48^, ^49^ Thus, with the reduction of CTLA‐4 and TIM‐3 expression, the function of Treg was clearly impaired, meanwhile the significant drop of IL‐10 in serum also gave strong support for it, suggesting lenvatinib might affect immune to some extent to improve the immune status of patients with HCC.

So, based on the result above, we constructed a novel index CTL/Treg ratio to evaluate the prognosis of patients with HCC after lenvatinib treatment, which could effectively reflect whether the patient's immune system was suppressed or not. The median of CTL/Treg ratio was used as a clinical cutoff value. We found patients with high CTL/Treg ratio (>2.96) had better clinical response. In addition, the Kaplan–Meier survival curve also indicated the CTL/Treg ratio had great discrimination ability as a prognosis predictor for PFS and OS. The reason might be that high CTL/Treg ratio suggesting the rebalance of the immune status with activation of CTL while suppression of Treg, which perform high antitumor capacity. On the other hand, we also found the correlation between CTL/Treg ratio and AFP, suggesting low CTL/Treg ratio might reflect the great damage of hepatocytes as well.

However, there were still remain some limitations in our study. First of all, the number of patients recruited in the research was small, the follow‐up period was short as well. Second, most patients in China have hepatitis B virus–positive background, which is quite different from the population in USA, Japan, Canada, and Europe. Therefore, the hypothesis we proposed needs to be further validated in larger and different patients from other geographic areas to explode the true relationship between immune cells and lenvatinib. Third, we found that lenvatinib could influence the number of immune cells and regulated the expression of immune checkpoint receptors on them, but the specific mechanism behind is still unclear, so further investigation into the underlying mechanism might provide a new sight of this medicine in treating HCC.

To our knowledge, this is the first research to demonstrate the immunomodulatory activity of lenvatinib in patients with HCC. Our results confirmed that lenvatinib is capable of improving patients’ immune status, saving the effector cells from exhaustion status and inhibiting the number and function of immunosuppressive cells. The novel index CTL/Treg ratio qualifies as a predictor for the outcome of patients with lenvatinib therapy, which is easy determination and low cost.

## CONFLICT OF INTERESTS

The authors declare that there are no conflict of interests.

## ETHICS APPROVAL

Ethics approval was obtained from the Research Ethics Committee of Zhongshan Hospital, Fudan University. Informed consent was obtained from each patient.

## Supporting information

Table S1

## Data Availability

The raw data will not be shared. All potential findings based on raw data analysis are presented in the manuscript or additional files.
