# Supplementary material for: The immunomodulatory activity of lenvatinib prompts the survival of patients with advanced hepatocellular carcinoma
Source: Cancer Med. 2021 Oct 4;10(22):7977–87. doi: 10.1002/cam4.4312 (PMC8607247; doi:10.1002/cam4.4312)
Supplement: Supplementary file 1 — Table S1 [file CAM4-10-7977-s001.docx]

Supplement Table 1. The Treatment response rate for HCC to Lenvtinib

| Response category | Lenvtinib |
| --- | --- |
|  |  |
| Complete response | 0(0%) |
| Partial response | 18(38.29%) |
| Stable disease | 24(51.06%) |
| Progressive disease | 5(10.63%) |
| Disease control rate | 42(89.36%) |
